# Occupational differences in COVID-19 hospital admission and mortality between women and men in Scotland: A population-based study using linked administrative data.

**DOI:** 10.23889/ijpds.v7i3.1841

**Published:** 2022-08-25

**Authors:** Serena Pattaro, Nick Bailey, Chris Dibben

**Affiliations:** 1 University of Glasgow/Scottish Centre for Administrative Data Research (SCADR)

## Objectives

We aim to estimate occupational differences in COVID-19 hospital admission and mortality by sex. Occupations vary with respect to environmental factors that influence exposure to COVID-19 such as ventilation, social contact and protective equipment. Variations between women and men may arise because they occupy different roles within an occupation or from behavioural differences.

## Approach

We established an innovative linked data collection combining individual-level data from 2011 Census with health administrative records and household identifiers from Ordnance Survey’s Unique Property Reference Number (UPRN). Using data for a cohort of approximately 1.7 million Scottish adults aged 40 to 64 years, we analysed occupational differences in COVID-19 hospital admission and mortality, during the period between 1 March 2020 and 31 January 2021. We estimated age-standardised incidence rates (ASIRs) for COVID-19 hospital admission and death per 100,000 adults, stratified by sex and occupation. Using Cox proportional hazards models, we estimated COVID-19 hospital admission and death risks, adjusting for socio-economic and pre-pandemic health factors.

## Results

Generally, women had lower ASIRs for COVID-19 hospital admission and mortality compared to men. For COVID-19 mortality, ASIRs were highest among women employed in elementary trades and related occupations (e.g. packers/canners). For hospital admission, high rates were observed among women working in caring personal services (e.g. nursing assistants/ambulance staff). Among men, ASIRs were highest among those in elementary services occupations (kitchen assistants/waiters) and taxi drivers, who also had the highest admission rate. After adjusting for pre-pandemic health factors, we observed lower death risks for women employed as health professionals, and those in associate professional and technical occupations (medical technicians/paramedics), compared to the baseline model. Lower adjusted admission risks were also observed for both women and men in professional occupations (e.g. in science/engineering and teaching/education). Among men, adjusted death risks remained elevated for large vehicle and taxi drivers, who also displayed high admission risks.

## Conclusion

Occupational differences in COVID-19 hospital admission and mortality between women and men may be explained by a mixture of social, workplace and behavioural factors. These need to be considered when designing intervention policies which aim to reduce infections in the workplace.

